# A patient with MELAS syndrome combined with autoimmune abnormalities: a case report

**DOI:** 10.3389/fneur.2023.1239664

**Published:** 2023-08-24

**Authors:** Mingmin Zhao, Chun Zuo, Hongyu Hao, Xing Xing, Lei Zhao, Na Li

**Affiliations:** ^1^Graduate School of Hebei North University, Zhangjiakou, Hebei, China; ^2^Neurological Intensive Care Unit, Hebei General Hospital, Shijiazhuang, Hebei, China; ^3^Hebei Provincial Key Laboratory of Cerebral Networks and Cognitive Disorders, Shijiazhuang, Hebei, China

**Keywords:** MELAS, mitochondrial encephalomyopathy, autoimmune abnormalities, antinuclear antibodies (ANA), antimetabolic glutamate receptor 5 encephalitis

## Abstract

**Background:**

Mitochondrial encephalomyopathy with lactic acidosis and stroke-like episodes (MELAS) is a group of maternally inherited disorders caused by mutations or deletions in mitochondrial genes with mitochondrial encephalomyopathy, lactic acidosis, and stroke-like episodes as the main clinical manifestations.

**Case presentation:**

We reported a 20-year-old female patient with MELAS syndrome combined with autoimmune abnormalities. She suffered from an intermittent headache in the right temporal region with no obvious cause, and then, after strenuous exercise in dance class, the headache became aggravated, accompanied by unresponsiveness, blurred vision, and diplopia. Her blood lactate levels were elevated, her antinuclear antibodies were positive, and the antimetabolic glutamate receptors 5 in her serum were positive. Brain DWI showed a hypertensive signal in the right temporo-parietal-occipital cortex and subcortical area. Brain MRS showed decreased NAA peak and increased Lac peak. Muscle biopsy showed myogenic damage, and the modified Gomori trichrome (MGT) staining showed ragged red fibers (RRF). A genetic study revealed a mitochondrial DNA A3243G mutation.

**Conclusion:**

Mitochondrial encephalomyopathy is a rare clinical condition; however, the association with autoimmune diseases is not yet clear and still needs further research and analysis.

## Introduction

Mitochondrial encephalomyopathy with lactic acidosis and stroke-like episodes (MELAS) is a group of maternally inherited disorders caused by mutation or deletion in the mitochondrial DNA (mtDNA) with mitochondrial encephalomyopathy (ME), lactic acidosis, and stroke-like episodes as the main clinical manifestations. The TL1m.3243A>G mutation in their mtDNA occurs in 80% of MELAS patients, with an incidence of ~8 to 236/1,00,000 in the population (1, 2). Antimetabolic glutamate receptor 5 (mGluR5) encephalitis is a rare autoimmune antibody-mediated disease of the central nervous system, it was first identified in two cases of limbic encephalitis (Ophelia syndrome), and similar case reports are uncommon (3, 4). Antinuclear antibodies (ANA) can be seen in a variety of diseases, and high titers of ANA can highly suggest the diagnosis of autoimmune disease (5). Therefore, this study reported a case of MELAS syndrome combined with positive anti-mGluR5 antibodies in serum and positive ANA in a patient with autoimmune abnormalities, thus raising awareness of these diseases.

## Case presentation

**Figure 1:**
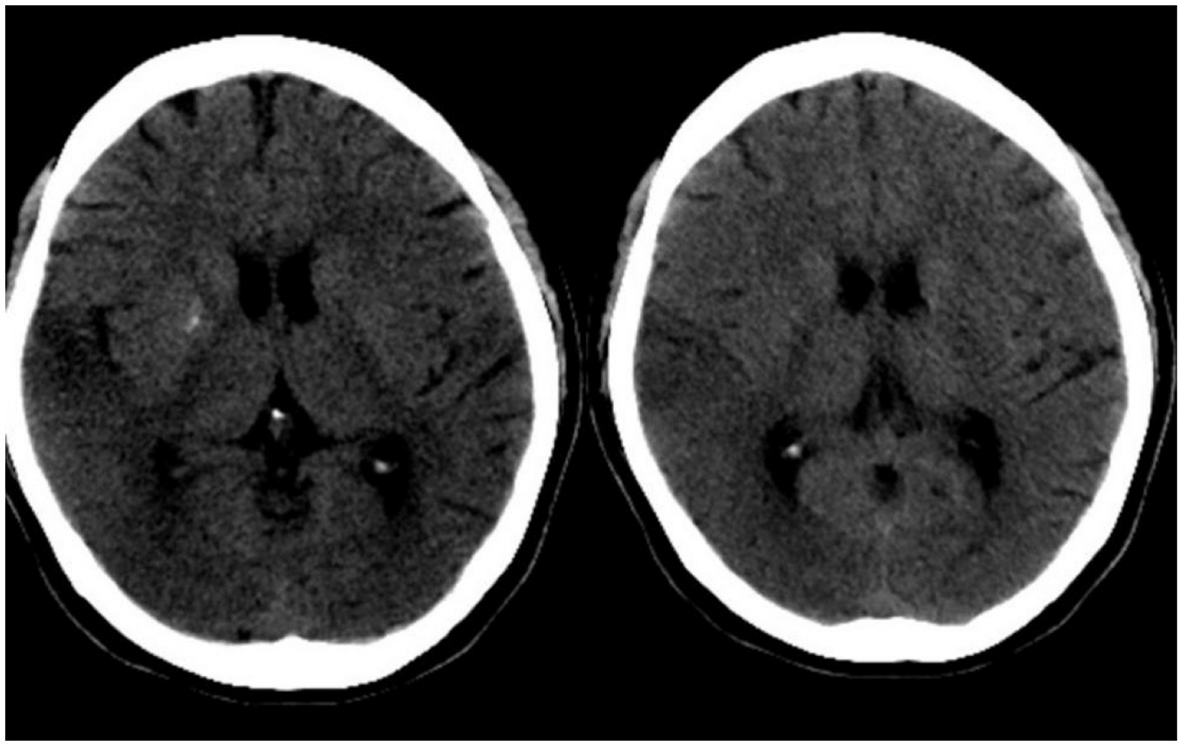
Cranial CT of the patient. On the day of the episode, cranial CT showed low density in the right temporo-parietal lobe and symmetric calcification in bilateral basal ganglia areas.

A 20-year-old woman was admitted to our hospital (April 2023) with complaints of intermittent headache in the right temporal region with no obvious cause, and then, after strenuous exercise in the dance class, the headache became aggravated, accompanied by unresponsiveness, blurred vision, and diplopia. Cranial CT (Figure 1) showed low density in the right temporo-parietal lobe and symmetric calcification in bilateral basal ganglia areas. The patient had no similar family history. She had cold 2 or 3 days ago. She had type 2 diabetes for 2 years, taking metformin hydrochloride extended-release tablets orally, without monitoring for blood glucose, “cerebral circulation insufficiency” for 1 year, with details unknown and no sequela, and bilateral hearing loss for more than 1 year, wearing a pair of hearing aid.

On examination, the patient was drowsy and unresponsive, with slightly slurred speech, and uncooperative on physical examination. Pupils were equal, round, and sensitively reacted to light. The face was symmetric with normal eye closure and a smile. The tongue was midline with normal movements. The muscle strength of the extremities was grade V. Muscle tone and reflexes were normal. Sensory and ataxic movements were unable to be detected. Pathologic reflexes were negative. The neck was supple, and there were no signs of meningeal irritation. After 1 day of this admission, the patient presented with two convulsive seizures. She yelled and screamed, and was confused, irritated, and uncooperative on physical examination. The left eye abduction is limited. The rest of the physical examination was unchanged. The patient is 150 cm tall and 37 kg weight, with a BMI of 16.44. Her mother said the patient had poor academic performance.

**Figure 2:**
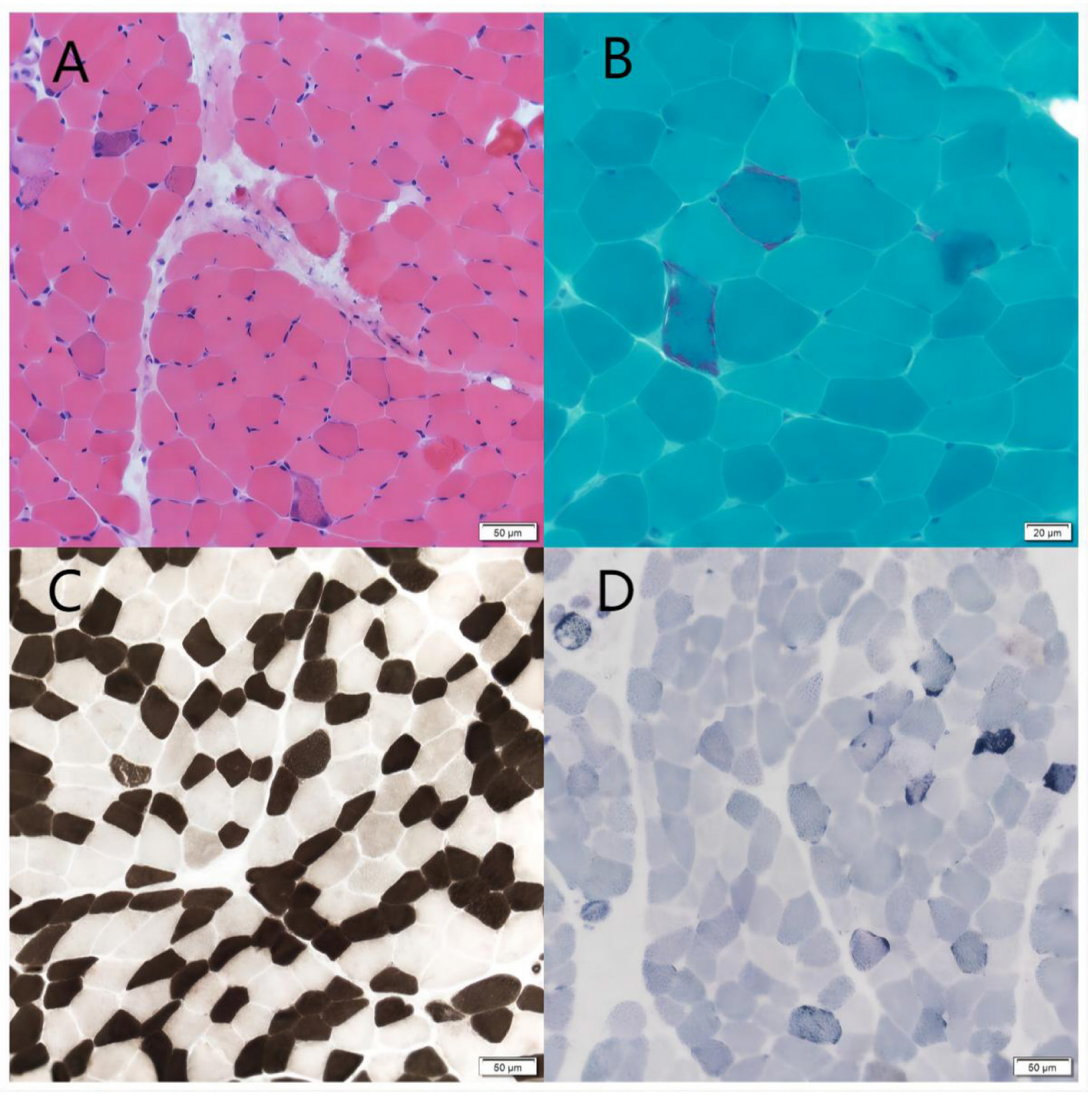
Muscle biopsy. **(A)** HE staining showed a mild hyperplasia of connective tissue of muscle bundles. Scale bars: 50 μm. **(B)** MGT staining showed ragged red fibers. Scale bars: 50 μm. **(C)** ATPase 10.2 staining showed the two muscle fibers were basically in a mosaic distribution. Scale bars: 50 μm. **(D)** SDH staining showed some muscle fibers were deeply stained or had deeply stained edges. Scale bars: 50 μm.

**Figure 3:**
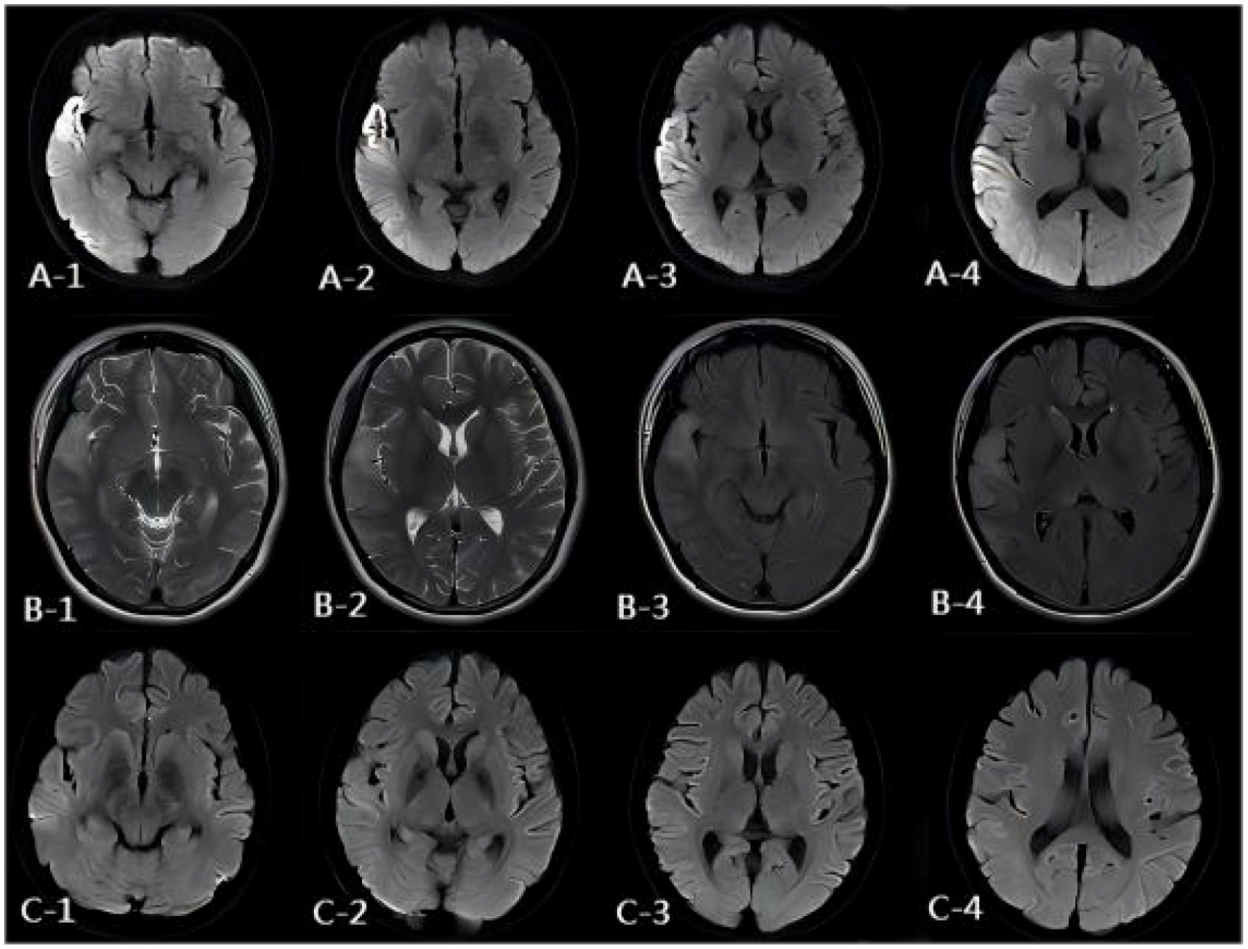
MR images of the patient. **(A)** DWI image of the onset period. DWI showed a hypertensive signal in the right temporo-parietal-occipital cortex and subcortical area. **(B)** DWI image of the onset period, 3 days after onset. MRI showed brain parenchymal swelling in the right temporoparieto-occipital lobe, abnormal signals of FLAIR, and T2WI of the right temporoparieto-occipital cortex and subcortical and right thalamus. **(C)** DWI image of the onset period, 13 days after onset. DWI showed the hypertensive signal in the right temporo-parietal-occipital cortex and subcortical area had disappeared.

**Figure 4:**
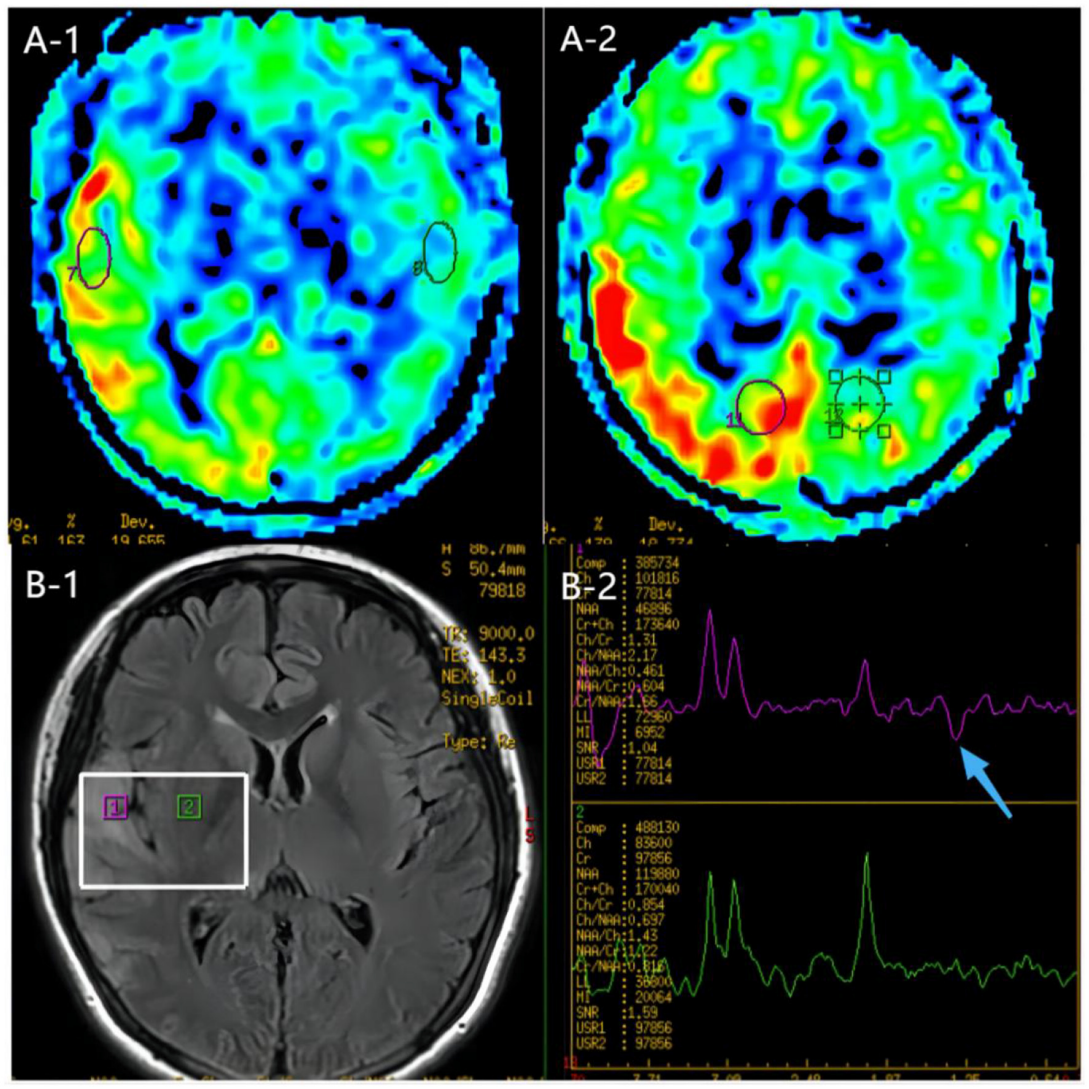
MR images of the patient. **(A)** ASL images of the onset period, 3 days after onset. ASL showed hyperperfusion in the right temporoparieto-occipital lesion area compared to the contralateral side. **(B)** MRS image of the onset period, 3 days after onset. MRS image showed decreased N-acetyl aspartate (NAA) peak and increased lactate peak, and there was a double inverted lactate peak at 1.31.ppm (blue arrow).

**Figure 5:**
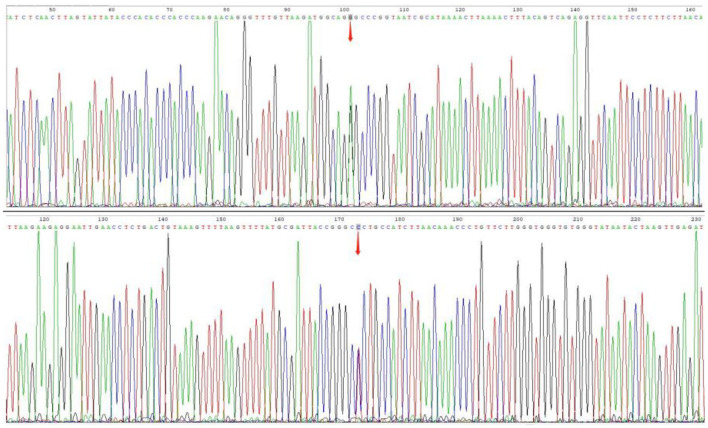
Gene mutations. Sanger sequencing for m.3243A>G mutations, and the level of heterogeneity was ~53.73% in her peripheral blood.

The patient's plasma lactate level was 14.77 mmol/L, and her ANA was positive (1:1000, normal range: negative), while other measurements, such as complete blood count, liver and kidney function tests, antiphospholipid antibodies, lupus anticoagulant, blood homocysteine, anti-cyclic citrullinated peptide antibodies (ACCP), and anti-keratin antibodies (AKA), were all normal. Her anti-mGluR5 was positive (1:10, normal range: negative), and the cerebrospinal fluid (CSF) autoimmune antibody test was normal. A CSF analysis showed a normal cell count, acid-fast staining, ink staining, protein, and glucose levels. Muscle biopsy showed myogenic damage, and the modified Gomori trichrome (MGT) staining showed ragged red fibers (RRF) (Figure 2). The staining of periodic acidic Schiff (PAS), oil red O(ORO), and non-specific esterase (NSE) was normal. Electroencephalogram (EEG) diffused low-to-middle amplitude slow waves, combined with low-amplitude fast activity, and normal EEG rhythm disappeared during awake and sleep states. Cardiac ultrasound, urinary ultrasound, lower extremity deep vein ultrasound, inferior vena cava, and thoracic ultrasound were normal. Brain diffusion-weighted imaging (DWI) revealed a hypertensive signal in the right temporo-parietal-occipital cortex and subcortical area (Figure 3). Magnetic resonance angiography (MRA) was normal. Brain magnetic resonance spectroscopy (MRS) showed decreased N-acetyl aspartate (NAA) peak and increased lactate peak, and there was a double inverted lactate peak at 1.31 (Figure 4). Brain magnetic resonance imaging (MRI) indicated brain parenchymal swelling in the right temporoparieto-occipital lobe and abnormal signals in the right temporoparieto-occipital cortex and subcortical and right thalamus. Arterial spin labeling (ASL) indicated hyperperfusion in the right temporoparieto-occipital lesion area compared to the contralateral side (Figure 4). A genetic study revealed a mitochondrial DNA A3243G mutation, and the level of heteroplasmy was ~53.73% in her peripheral blood (Figure 5).

The patient was diagnosed with MELAS syndrome by gene testing. She received the treatment with propofol (0.5 g/time) sedation, levetiracetam (1 g/day), and clonazepam tablets (2 mg/day) for seizure control, adenosine triphosphate disodium (ATP) (120 mg/day) to improve cellular energy metabolism, L-arginine (15 g/day) to treat stroke-like episodes, coenzyme Q10 (30 mg/day) to scavenge free radicals, and sodium bicarbonate (3 g/day) to reduce lactic acid and correct acidosis. Then, the patient was seizure-free for 5 days but still had psychotic symptoms. After 13 days of treatment, the acidosis was corrected, lactic acid was reduced to 2.74 mmol/L, and brain DWI showed the hypertensive signal in the right temporo-parietal-occipital cortex and subcortical area disappeared (Figure 3B). Then, she was discharged.

## Discussion

ME is a group of diseases with pathophysiological mechanisms based on mtDNA or nuclear DNA (nDNA) defects, resulting in oxidative phosphorylation dysfunction of the mitochondrial respiratory chain and insufficient ATP production, thus causing muscle and central nervous system dysfunction. It is one of the most common maternally inherited mitochondrial diseases. The disease can involve multiple systems and may manifest clinically as muscle weakness, cranial and peripheral nerve damage, seizures, and metabolic abnormalities. ME can be divided into four subtypes: mitochondrial encephalomyopathy with lactate acidosis and stroke-like episodes (MELAS), myoclonic epilepsy with ragged red fibers (MERRF), Kearns–Sayre's syndrome (KSS), and mitochondrial neurogastrointestinal encephalomyopathy (MNGIE). MELAS syndrome, which occurs most frequently, accounting for 80% of ME cases, onsets usually between 2 and 40 years of age. It has broad manifestations including stroke-like episodes, dementia, epilepsy, lactic acidemia, myopathy, recurrent headache, hearing impairment, diabetes, and short stature (6). Retinopathy and gastrointestinal dysfunction may be accompanied in a few patients. The study focuses on MELAS syndrome with autoimmune dysfunction.

MELAS syndrome was first described as a unique mitochondrial disorder in 1984, and the pathogenesis has not been elucidated. It is currently considered to be the result of the interaction of multiple factors such as a genetic mutation in mtDNA or nDNA, which in turn leads to mitochondrial structure and functional disorders, energy deficiency, vasculopathy, and nitric oxide (NO) deficiency (7). Studies have shown that ~80 to 90% of MELAS patients have m.3243 A>G mutation of tRNALeu(UUR) gene, which leads to the obstruction of the translation and synthesis of mitochondrial proteins and affects the synthesis of mitochondrial electron transport chain (ETC) complex subunits, which in turn causes mitochondrial dysfunction and impaired energy production. The inability of dysfunctional mitochondria to generate sufficient ATP to meet the energy needs of various organs results in multi-organ dysfunction and respondent clinical symptoms, such as dysfunction of high energy-demanding organs including the central nervous system, kidneys, and endocrine system observed in MELAS syndrome. In addition, energy deficiency can stimulate mitochondrial proliferation in smooth muscle and endothelial cells of small blood vessels, which leads to impaired blood perfusion in the microvasculature of multi-organ, and it is most significantly associated with stroke-like episodes in MELAS syndrome (6). Citrulline is converted to arginine by argininosuccinate synthase and argininosuccinate lyase, and arginine is catalyzed by nitric oxide synthase (NOS) to produce NO and citrulline. Mitochondrial proliferation in vascular endothelial cells leads to endothelial dysfunction and impaired NO synthesis and secretion. Excessive production of reactive oxygen species after ETC injury triggers oxidative stress, leading to an increase in asymmetric dimethylarginine, which inhibits the activity of nitric oxide synthase (NOS) and leads to the decrease of NO. Cytochrome C oxidase can inhibit the production of NO in the late stage of mitochondrial proliferation, and oxidative stress can induce the conversion of NO to active nitrogen, both of which lead to NO deficiency. A deficiency of NO, which has the function of maintaining vascular smooth muscle relaxation, can lead to impaired microvascular perfusion in different organs and various complications such as stroke-like episodes (8).

The patient was a young 20-year-old woman who presented with stroke-like episodes as her primary symptom. She was short in stature and had a history of diabetes and bilateral hearing loss. Brain MRI indicated brain parenchymal swelling in the right temporoparieto-occipital lobe and abnormal signals in the right temporoparieto-occipital cortex and subcortical and right thalamus. She had hyperlactatemia. Brain MRS showed decreased NAA peak and increased lactate peak, and there was a double inverted lactate peak at 1.31. Muscle biopsy showed myogenic damage, and the MGT staining showed RRF. A genetic study revealed a mitochondrial DNA A3243G mutation, the level of heterogeneity was ~53.73% in her peripheral blood, and MELAS syndrome was diagnosed. She was easily misdiagnosed with ischemic cerebrovascular disease. However, MRA did not indicate stenosis or occlusion of the corresponding cerebral responsible vessels. Therefore, we suspected the diagnosis of ischemic cerebral infarction and other diseases, such as MELAS.

The mutated single nucleotide in this patient was located in the mitochondrial tRNALeu (UUR), and MT-TL1 contained mtDNA base pairs 3230–3304. The pathogenic point mutation positions of MT-TL1 were identified as 3242 G>A,3243 A>G,3249 G>A,3250 T>C,3251 A>G,3256 C>T,3260 A>G,3271 T>C,3274 A>C,3290 T>C,3303 C>T, etc. Mutations in this gene can cause mitochondrial disease, disrupting the function of the leucine-transporting tRNA in the mitochondria, thus blocking the translation and synthesis of mitochondrial protein, and leading to mitochondrial dysfunction and insufficient energy synthesis. The mutation was detected in numerous patients with mitochondrial disease, with clinical manifestations including MELAS, PEO, mitochondrial diabetes, deafness, and headache (9, 10). Leucine is the only amino acid in the skeletal and cardiac muscles which regulates protein turnover and nitrogen utilization in the tissue, thereby promoting protein synthesis in the organism. In addition, its metabolite β-hydroxy-β-methylbutyric acid (HMB) can regulate protein metabolism and relieve fatigue. Mutations in this gene result in impaired energy metabolism and complex clinical symptoms. Generally, the clinical manifestations of mitochondrial disease often involve multiple systems and organs, it can be variable with age, and it is often accompanied by characteristic histochemical and biochemical abnormalities. MELAS is known to involve the nervous system, skeletal and cardiac muscles, kidney, liver, and endocrine system (6). There are no reports of autoimmune system involvement.

The antimetabolic glutamate receptor 5 (mGluR5) belongs to the first group of G protein-coupled receptors; mGluR5 is found in the postsynaptic terminals of neurons and microglia cells; it is widely expressed in the central nervous system and is mainly distributed in the striatum, cerebral cortex, hippocampus, amygdala, and other regions of expression (11). It was initially reported as neoplasm-associated encephalitis; however, anti-mGluR5 encephalitis can also occur in the absence of tumors. Anti-mGluR5 encephalitis is a rare disease, and no cases have been reported in Asia. Clinical manifestations may include psychiatric disorders, cognitive impairment, epilepsy, sleep disturbance, movement disorder, and neurological damage to the brain, with large individual differences (12). Positive anti-mGluR5 antibodies in the serum and the cerebrospinal fluid are the critical diagnostic criteria for anti-mGluR5 encephalitis. The diagnosis of anti-mGluR5 encephalitis was not established in this patient who was positive for anti-mGluR5 antibodies in serum only. In addition, there were no further behavioral/personality changes after the resolution of seizure symptoms.

ANA is a non-specific autoantibody that targets various components of eukaryotic cells as target antigens. In autoimmune diseases, activated lymphocytes may initiate the production of ANA. The nucleus becomes an important target of autoimmune response, and after activation of lymphocytes caused by various factors, nuclear antigens are changed and may express new antigenic determinants, or cryptic antigenic determinants (such as the nuclear membrane of the nucleus, intranuclear chromatin, non-histone proteins, and various riboprotein particles composed of ribonucleic acid and related proteins) are exposed, breaking auto-T cell resistance and assisting B cells to produce antibodies and produce ANA. ANA is a group of autoantibodies produced against DNA, RNA, proteins, or molecular complexes of these substances in the cell nucleus. These antibodies damage the internal structure of the cell nucleus, specifically attacking the cell DNA and its internal components, causing the death of the nucleus and cells, and when the number of cell death reaches a certain level, the organ tissue is seriously damaged, and the organ eventually fails. ANA can be seen in a variety of diseases, especially connective tissue diseases, and is often used as an indicator for diagnosis, disease determination, and efficacy observation of connective tissue diseases. It can also be positive in non-connective tissue diseases, infectious diseases, or tumors, and high-titer ANA is highly suggestive of autoimmune diseases and is an extremely important clinical screening indicator for autoimmune diseases (5).

More and more evidence has shown that mitochondria are the center point of the immune system and play an important role in the activation of the immune system and the regulation of immune metabolism (13). Mitochondria activate the immune system mainly based on the following three molecular mechanisms: (1) mitochondrial component activates the inflammatory vesicle NOD-like receptor protein 3 (NLRP3) to participate in the inflammatory response and the immune response of the organism; (2) mitochondrial component of foreign antigens becomes intracellular antigens through the mitochondrial permeability transition pore; (3) mtDNA activates toll-like receptor 9 (TLR-9), and cyclic GMP-AMP synthase/stimulator of interferon gene (cGAS/STING) signaling induces the body's inflammatory response and immune response. The metabolic regulation of immune cells is important in the survival, proliferation, and activation of immune cells (14, 15). Mitochondria play an essential role in metabolic regulation by influencing glucose oxidation and fatty acid, amino acid, and hormone biosynthesis, which depends on the stability of mitochondrial dynamics and function. In addition, mitochondrial reactive oxygen species (mtROS), which are produced along with oxidative phosphorylation (OXPHOS) and electron transport chain (ETC), can modulate a wide range of immune environments and pathologically damaging factors. ROS are involved in a variety of diseases, including autoimmune diseases. Therefore, it is undeniable that the immune system of this patient was compromised by mitochondrial dysfunction, and at this time, we only observed an abnormality in the ANA, which is used as a primary screening indicator for immune system disorders, and long-term follow-up is needed to determine whether other disorders have occurred.

There is no cure for MELAS syndrome, but symptomatic treatment to reduce clinical symptoms and improve prognosis is the main focus. Stroke-like episodes, migraine, cardiac lesions, diabetes, sensorineural deafness, and seizures can be treated with its regular standard regimen. Valproic acid should be avoided for the treatment of seizures because of its adverse effects on mitochondrial function, which can induce or aggravate seizures (16). Recent studies have shown that the novel therapy drugs such as L-arginine and nicotinamide adenosine dinucleotide (NAD) modulators KL1333 can be used in the treatment of MELAS syndrome, the former being beneficial in the treatment and prevention of stroke-like episodes, and the latter improving mitochondrial production and function, which can also be used in the treatment of MELAS syndrome (17, 18). Propofol infusion syndrome (PRIS) is a rare syndrome associated with the high-dose and long-term infusion of propofol because of the widespread use of propofol, with crucial features including cardiac failure accompanied by progressive arrhythmias, metabolic acidosis, and renal failure. Studies suggest that the primary mechanism may be due to impaired β-oxidation of free fatty acids in the mitochondria and inhibition of electron transport in the mitochondrial respiratory chain, which in turn inhibits intracellular energy production (19). This patient was treated with a small dose of propofol [2 mg/(kg-h)], and propofol sedation was discontinued within 24 h of the first seizure; there was no exacerbation of pre-existing metabolic acidosis, and no symptoms of heart failure or progressive arrhythmia occurred during treatment. Therefore, the use of propofol sedation in patients with mitochondrial encephalomyopathy should be used carefully to avoid propofol infusion syndrome.

The patient was positive for antinuclear antibodies and positive for anti-mGluR5 in the serum, and no anti-mGluR5 antibodies were found in CSF, so it cannot be correlated with the patient's abnormal mitochondrial function, and there has been no reported case of co-morbidity or superposition of mitochondrial encephalopathy with autoimmune-related diseases. Therefore, the diagnosis of autoimmune-related disease in this patient was not clear at this time, but it was undeniable that the patient's autoimmune function was impaired, and the development of other immune system diseases with the progression of the disease over time requires long-term follow-up. We gave her discharge instructions related to supportive therapy, including dietary therapy and exercise therapy. In addition, the possibility of immune system involvement in mitochondrial encephalopathy and its pathogenesis still needs to be further explored.

## Data availability statement

The datasets presented in this article are not readily available because of ethical and privacy restrictions. Requests to access the datasets should be directed to the corresponding author.

## Ethics statement

The studies involving human participants were reviewed and approved by the Ethics Committee of the Hebei General Hospital. Written informed consent to participate in this study was provided by the patients/participants. Written informed consent was obtained from the individual(s) for the publication of any potentially identifiable images or data included in this article.

## Author contributions

MZ performed the data analyses and wrote the manuscript. CZ and HH contributed significantly to the analysis and manuscript preparation. LZ and XX contributed to the conception of the study. NL was responsible for the final revision of the study. All authors contributed to the article and approved the submitted version.
